# Security-oriented steganographic payload allocation for multi-remote sensing images

**DOI:** 10.1038/s41598-024-55474-y

**Published:** 2024-02-28

**Authors:** Tian Wu, Xuan Hu, Chunnian Liu

**Affiliations:** Digital Literacy and Skills Enhancement Research Center, Jiangxi Province Philosophy and Social Science Key Research Base, School of Public Policy and Administration, Nanchang University, Nanchang, 330031 China

**Keywords:** Computer science, Information technology

## Abstract

Multi-image steganography, a technique for concealing information within multiple carrier mediums, finds remote sensing images to be particularly apt carriers due to their complex structures and abundant texture data. These characteristics bolster the resilience against steganalysis and enhance steganographic capacity. The efficacy of multi-image steganography hinges on the diplomatic strategy of cover selection and the meticulous allocation of the payload. Nevertheless, the majority of current methods, which are empirically formulated, predominantly focus on the texture complexity of individual images, thereby potentially undermining overall security. This paper introduces a security-oriented approach to steganographic payload allocation for multiple remote sensing images aimed at fortifying the security of multi-image steganography. Our primary contributions include employing a steganalysis pre-trained network to quantify texture complexity in remote sensing cover images, directly correlating it with security. Additionally, we have developed an adaptive payload allocation strategy for multiple images, which embeds a payload proximate to each image’s maximal steganographic capacity while concurrently ensuring the security of the embedding process. Experimental results corroborate that our methodology excels in cover selection and payload allocation and achieves better undetectability against modern steganalysis tools.

## Introduction

Image steganography is a technique to conceal secret information within the cover object without arousing the attacker’s suspicion^1,2^. Early steganography approaches focused on minimizing the number of modifications to the cover image^3,4^. With the introduction of Syndrome-Trellis Codes (STC) for minimizing additive distortions^5^, steganographers only need to be concerned with the design of the steganographic cost function^6,7^, as STC assists in embedding the secret message in a safe location while ensuring minimal global distortion. Currently, researchers are incorporating techniques such as adversarial learning and reinforcement learning into steganography to foster enhanced security^8,9^. However, given the explosive growth of Internet images and the capacity limitations of single-image steganography, steganographers should adopt appropriate strategies to allocate the payload rationally across multiple carriers in practical applications, especially when transmitting large volumes of secret messages.

Multi-image steganography, often called batch steganography, necessitates that steganographers rationally allocate and embed substantial amounts of secret information into appropriate carriers while maintaining optimal undetectability. Therefore, multi-image steganography has two core issues: cover selection and payload allocation. Regarding cover selection, cover images in early multi-carrier steganography were often selected empirically based on metrics such as PSNR and stego image modifications^10^, which posed challenges to achieving improved performance. Subsequent researchers advocated for selecting the cover image based on texture complexity, leading to better performance. Consequently, the focus in cover selection shifted to measuring image texture complexity^11,12^. Additionally, a combination of embedded distortion with either processed distortion^13^ or image similarity^14^ has been suggested for selecting cover images. However, despite these advancements, these methods remain empirical and fail to establish a direct link between carriers and their security.

On the aspect of payload allocation, Ker A. D et al. initially introduced five embedding methods for allocating payloads in multiple cover images. Their research^15^ demonstrated that a smaller number of stego images results in enhanced security performance. These methods include maximal greedy, maximal random, linear, even number, and square root methods^16^. However, these methods are solely applicable to non-adaptive steganography, thereby restricting their practical applicability. For adaptive steganography, three near-optimal strategies have been proposed: IMS (Image Merge Sender)^17^, DeLS (Detectability Limited Sender)^18^, DiLS (Distortion Limited Sender)^19^. These strategies employ a hypothesis-testing framework to optimize the security and efficiency of multi-image steganography. Nevertheless, the complexity of their computational processes makes them challenging to implement in practical scenarios. Subsequently, Wang et al.^20^ demonstrated that the first-order derivative of steganographic distortion in a single cover monotonically increases, leading to the design of an algorithm that combines cover selection and payload allocation. Hu et al.^21^ proposed a linear approach to payload allocation based on the intrinsic energy of JPEG images. Recently, Liao et al.^11^ proposed an adaptive multi-image steganographic payload allocation method based on image texture complexity.

In practical applications, remote sensing images are particularly well-suited for multi-image steganography due to the inherent diversity and complexity of these images, encompassing varying land cover and urban structures, thereby offering excellent concealment for steganographic content, making detection more challenging. This characteristic is crucial in steganography as it enhances the security and capacity of the embedded information^22^. Additionally, the dynamic nature of remote sensing technology, which continuously generates a vast amount of data, offers a comprehensive and constantly evolving range of potential covers for multi-image steganography, significantly enhancing its applicability and effectiveness. Therefore, remote sensing images are ideal for executing complex and secure steganographic operations.

To forge a connection between multi-image steganography and security, we introduce an innovative framework that allocates payloads across multiple remote sensing images. Our framework encompasses three aspects: cover type, cover selection, and embedding strategy. It entails two steps when utilizing remote sensing images as covers: cover selection and payload allocation. For cover selection, we employ a novel method under multiple steganography algorithms to identify the most secure cover images from a pool of remote sensing images, eschewing the conventional image complexity-based method. For payload allocation, we devise a novel embedding strategy that enables the embedding of a payload close to each image’s maximum steganographic capacity, simultaneously minimizing the stego images’ detectability. Thus, we can transmit more secret information with fewer images in real-world scenarios.

In summary, this article makes the following contributions:

- We proposed a security-oriented payload allocation method for multi-remote sensing images, which minimizes the number of selected cover images and makes full use of the maximum steganographic capacity, significantly enhancing the security of multi-image steganography.
- A cover selection method is proposed to establish a direct connection between image selection and image security, ensuring that the selected cover image is highly resistant to steganalysis.
- A payload allocation strategy based on a deep steganography pre-training model has been developed, which employs a deep steganalysis model as a discriminator, enhancing the security of steganography embedding and improving the accuracy of payload allocation.
- Experimental results show that our proposed multi-image steganography method overall performs better than the state-of-the-art methods and does not require extensive computation, which makes it very suitable for applications in real-world scenarios.

## Related works

This section concisely reviews the related works about state-of-the-art cover selection and payload allocation strategy.

### Cover selection

In the early stages of multi-image steganography, empirical criteria such as PSNR and cover image modification amount^23^, as well as texture complexity measures like residual, image potential state, and image fluctuation^24^, were relied upon. However, these methods have limitations in terms of performance, computational cost, accuracy, or robustness. This paper presents a novel texture complexity measure derived from the image residual, which is calculated using the WOW steganography algorithm^25^. The WOW algorithm is known for its high security and low distortion. It calculates the residuals in three directions: horizontal, vertical, and diagonal. The image residual *R* is defined as follows:

1 $$\begin{aligned} R=\sum _{k=1}^3 \sum _{i=1}^{n_1} \sum _{j=1}^{n_2} \mid r_{i, j}^{(k)}\mid \end{aligned}$$

where $r_{i, j}^{(k)}$ is the residual value of the pixel at position (*i*, *j*) for an image of size $n_{1} \times n_{2}$. To additionally augment the texture complexity measure, Sobel filter was applied to derive the image residual and obtain the spatial information (SI) of each pixel, as delineated below:

2 $$\begin{aligned} S I_r=\sqrt{s_h^2+s_v^2} \end{aligned}$$

where $s_h$ and $s_v$ are the horizontal and vertical Sobel kernel filtering results^26,27^. The resulting SI image is a grayscale image that reflects the texture complexity of the cover image.

The complexity of an image is quantifiable by its SI. The SI of an image with *M* pixels relies on three metrics: the mean, the root mean square, and the standard deviation. A fuzzy set, representing the image complexity, is generated using these metrics, as defined below:

3 $$\begin{aligned} \left\{ \begin{array}{l} S I_{\text{ mean } }=\frac{1}{M} \sum S I_r \\ S I_{\text{ rms } }=\frac{1}{M} \sum S I_r^2 \\ S I_{\text{ stdev } }=\sqrt{\frac{1}{M} \sum S I_r^2-S I_{\text{ mean } }^2} \end{array}\right. \end{aligned}$$

Liao et al.^11^ measured the texture complexity of an image by computing its two-dimensional (2D) entropy using the KerBohme high-pass filter. They applied the the filter $F_{KB}$ to image set *X* to derive the residual set $X'$, as illustrated below:

4 $$\begin{aligned} \left\{ \begin{array}{ll} X^{\prime } =X \otimes F_{K B} \\ F_{K B} =\left[ \begin{array}{ccc} -1 &{} 2 &{} -1 \\ 2 &{} -4 &{} 2 \\ -1 &{} 2 &{} -1 \end{array}\right] \end{array}\right. \end{aligned}$$

They proceeded to calculate the grey scale covariance matrix *P* of the residual $x'$ for each pair of pixels ($a_1$, $b_1$) and ($a_2$, $b_2$) with values *u* and *v*, distance *d*, and angle $\theta \in \left\{ 0^{\circ }, 45^{\circ }, 90^{\circ }, 135^{\circ }\right\}$, as detailed below:

5 $$\begin{aligned} P(u, v, d, \theta )=&\xi \{ \left( a_1, b_1\right) ,\left( a_2, b_2\right) \mid x_i \left( a_1, b_1\right) =u, \mid x_i\left( a_2, b_2\right) =v, \text{ s.t. } \left. \mid \left( a_1, b_1\right) -\left( a_2,b_2\right) \mid =d, \right. \nonumber \\&<\left( a_1, b_1\right) ,\left( a_2, b_2\right) >=\theta \} \end{aligned}$$

where 1 < $a_{1}$, $a_{2}$ < $n_{1}$, 1 < $b_{1}$, $b_{2}$ < $n_{2}$, and $\xi \{W\}$ is the number of elements in the set that satisfy *W*. Finally, they determined the 2D entropy *h* of the image by averaging the entropy $h_i(\theta )$ across four directions as follows:

6 $$\begin{aligned} \left\{ \begin{array}{l} \tilde{h}_i =\frac{\sum _\theta h_i(\theta )}{4} \\ h_i(\theta ) =-\sum _u \sum _v P(u, v, d, \theta ) \log _2 P(u, v, d, \theta ) \end{array}\right. \end{aligned}$$

Wang et al.^13^ introduced a method to determine the optimal image for steganography by reducing the total distortion, comprising the steganographic distortion (caused by embedding hidden information) and the processing distortion (caused by image enhancement or other operations). They quantified the processing distortion using the MMD distance, expressed as follows:

7 $$\begin{aligned} \min _{ I \in \mathscr{I}} D(I) = \min _{I \in \mathscr{I}} (D_e(I) + D_p(I)) \end{aligned}$$

where $\mathscr{I}$ represents the set of available images, *D*(*I*) denotes the total distortion of image *I*, $D_e(I)$ is the steganographic distortion, and $D_p(I)$ represents the MMD distance between image *I* and a collection of clear images $\mathscr{J}$, defined below:

8 $$\begin{aligned} D_p(I) = \frac{1}{n^2} \sum _{i,j=1}^n k(I_i, I_j) - \frac{2}{nm} \sum _{i=1}^n \sum _{j=1}^m k(I_i, J_j) + \frac{1}{m^2} \sum _{i,j=1}^m k(J_i, J_j) \end{aligned}$$

where *n* and *m* are the number of images in $\mathscr{I}$ and $\mathscr{J}$ respectively, $k(\cdot , \cdot )$ is a kernel function. $I_i$ and $J_j$ are the images in $\mathscr{I}$ and $\mathscr{J}$ respectively.

In another work, Wang et al.^14^ proposed a novel image selection strategy for steganography grounded in SVD (singular value decomposition). They calculated the similarity between images using the SVD coefficients, subsequently integrating the similarity and the embedding distortion to identify the most suitable image. The similarity measure is as follows:

9 $$\begin{aligned} S_{ij}=\frac{\sum _{k=1}^n s_{ik}s_{jk}}{\sqrt{\sum _{k=1}^n s_{ik}^2}\sqrt{\sum _{k=1}^n s_{jk}^2}} \end{aligned}$$

where $S_{ij}$ is the similarity between the *i*-th and the *j*-th images, and $s_{ik}$ is the *k*-th singular value of the *i*-th image.

### Payload allocation strategy

Early stego payload allocation strategies typically employed five strategies: equal embedding strategy, linear strategy, root mean square strategy, maximum greedy strategy and maximum random strategy^11^. The fundamental concept of these strategies involves using all images or as few as possible to convey the message. However, these strategies fail to consider the image features and the distortion caused by data embedding, potentially affecting the security performance of steganography. Consequently, several adaptive payload allocation strategies focusing on the image texture features and distortion allocation have been proposed in recent years^28^. These strategies are designed to allocate payload among multiple images based on their texture complexity and distortion sensitivity, aiming to minimize steganalysis detectability. Assuming the number of cover images is *N*, the length of the secret message is *M*, and $c_i$ denotes the steganographic capacity of the image *i*, the payload $m_i$ of each image using the equal embedding, linear, and root-mean-square strategies is calculable as follows:

Equal Embedding Strategy:

10 $$\begin{aligned} m_i=\frac{M}{N} \end{aligned}$$

Linear Strategy:

11 $$\begin{aligned} m_i=\frac{c_i M}{\sum _{j=1}^N c_j} \end{aligned}$$

Root Mean Square Strategy:

12 $$\begin{aligned} m_i=\frac{\sqrt{c_i} M}{\sum _{j=1}^N \sqrt{c_j}} \end{aligned}$$

The maximum greediness and maximum randomness strategies share similarities but differ in their methods of selecting images for embedding secret messages. The maximum greediness strategy organizes the images in descending order of their potential, first opting for the most significant ones. This process persists until all the messages are embedded. Conversely, the maximum randomness strategy randomly selects images, disregarding their ability. This process entails embedding messages into randomly selected images until all are embedded. The following formula can express both strategies:

13 $$\begin{aligned} \left\{ \begin{array}{l} m_i=c_i, \quad i \in \{1,2, \ldots , I-1\} \\ m_i=M-\sum _{i=1}^{I-1} c_i, \quad i=I \\ m_i=0, \quad \text{ others } \end{array}\right. \end{aligned}$$

Wang et al.^20^ introduced a batch steganography strategy aimed at minimizing the total steganographic distortion of the selected images by equalizing the first-order derivatives of the distortion of individual images relative to the payload. They expressed the problem as follows:

14 $$\begin{aligned} \min _{\textbf{x}} \sum _{i=1}^n \left( \frac{\partial D_i}{\partial x_i} \right) ^{-\alpha } \end{aligned}$$

where $\textbf{x}$ represents the payload vector, $D_i$ denotes the distortion of the *i*th image, and $\alpha$ is a positive constant. Hu et al.^21^ subsequently introduced a novel JPEG batch steganography scheme that allocates the payload linearly according to the intrinsic energy of each image, a heuristic indicator of the high-frequency content. The payload allocation equation is:

15 $$\begin{aligned} x_i = \frac{E_i}{\sum _{j=1}^n E_j} \times x_{total} \end{aligned}$$

where $x_i$ represents the payload for the *i*th image, $E_i$ indicates its intrinsic energy, $x_{total}$ is the total payload, and *n* denotes the number of image.

Barring the equal embedding strategy, most existing batch steganography strategies require prior knowledge of the steganographic capacity, limiting them to less secure stego methods. To implement more secure adaptive steganography techniques, subsequent research has introduced three strategies based on different criteria: IMS, DeLS, and DiLS. The IMS strategy, an algorithm-based approach, merges all cover images into a single image, embeds it using a unified steganography algorithm, and then divides it back into multiple original images. This strategy effectively reduces the overall distortion and yields the best practical performance^29^. The DeLS and DiLS strategies, focusing on cover-image-based and distortion-based approaches, respectively, strive to maintain high stego security by equalizing each image’s KL divergence or distortion before and after steganography^30^. These three strategies can achieve near-optimal stego payload allocation but entail significant computational and memory costs in scenarios with multiple images^31^.

## Security-oriented multi-image adaptive payload allocation architecture for remote sensing images

**Figure 1:**
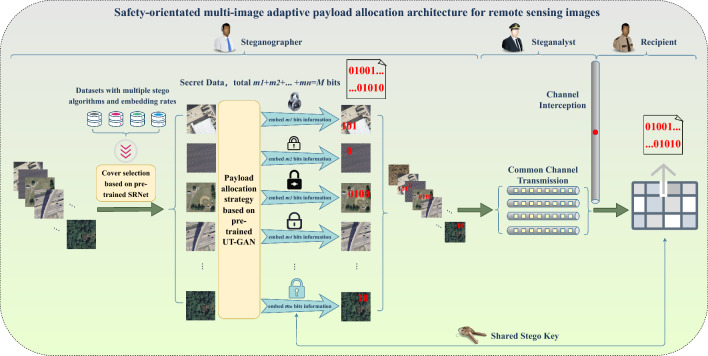
The security-oriented multi-image adaptive payload allocation architecture for remote sensing images.

The paper presents the security-oriented multi-image adaptive payload allocation architecture, as depicted in Fig. 1.

Employing two pre-trained models, we address the central challenges of multi-image steganography: cover selection and payload allocation. Initially, the pre-trained SRNet^32^ is employed to select covers from two image datasets. Following the acquisition of the selected cover images, the corresponding embedded payloads are allocated using the pre-trained UT-GAN^33^. The selected image is concealed using steganography with the aid of the pre-trained UT-GAN, achieving the required payload length. Ultimately, the resulting stego image is merged with a cover image and transmitted to the recipient through a shared channel. As an observer of multi-image steganography, one must scrutinize the multi-image steganography of various images shared by individuals on the public channel to determine their steganography identity.

### Cover selection based on a steganalysis pre-trained model

Pre-trained models, trained on large datasets like ImageNet^34^, are widely used in deep learning to enhance performance and generalization across various domains. This study investigates the applications of pre-trained models in image steganography and steganalysis, focusing on concealing and detecting secret messages in images. We explore recent advancements in pre-trained model-based steganography^35^ and steganalysis^36,37^, and propose a novel method to enhance steganography’s security.

Previous methods used filtering techniques to measure the texture complexity of cover images but overlooked the influence of different steganography algorithms or embedding rates^30^. Additionally, they failed to compare the texture complexity among different cover images, potentially compromising the steganographic system’s security. To address this, we introduce a novel cover selection method informed by a steganalysis pre-trained model capable of estimating the security of stego images under various embedding scenarios. This method effectively measures and ranks the texture complexity of cover images based on their suitability for steganography. We categorize two levels of security for multi-image steganography: image level security and individual level security. Image level security ensures that stego images remain indistinguishable from cover images using single-image steganalysis methods^38^. Individual level security assesses the degree of anomaly in each stego image before and after embedding, utilizing the LOF^39^ derived from the MMD distance^40^. The MMD distance, as shown in Eq. (16), is determined by the number of samples in each dataset and is independent of feature dimensionality. We emphasize that the MMD distance influences the individual-level security assessment, and increasing the number of unorganized cover images in multi-image steganography reduces the MMD value. Therefore, our cover selection method prioritizes image-level security.

16 $$\begin{aligned} {\text{MMD}}[\phi , \chi , \gamma ]=\left[ \frac{1}{n(n-1)} \sum _{i \ne j} \phi \left( x_i, x_j\right) +\phi \left( y_i, y_j\right) - \phi \left( x_i, y_j\right) -\phi \left( x_j, y_i\right) \right] ^{\frac{1}{2}} \end{aligned}$$

**Figure 2:**
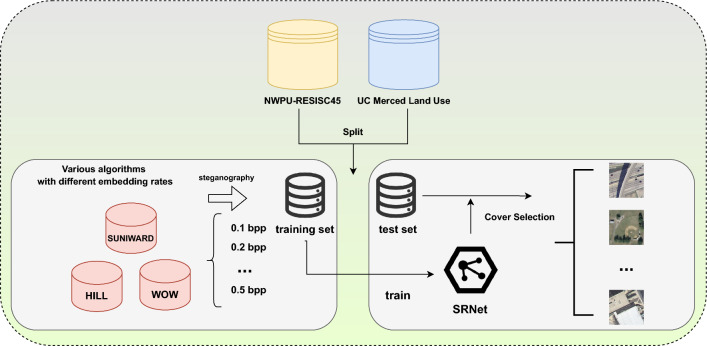
Visualisation of the process of training SRNet for cover selection.

To tackle the challenge of cover selection amid diverse stego algorithms and embedding rates, we aim to train SRNet, a highly efficient steganalysis network, on a dataset featuring a variety of steganography algorithms and embedding rates. We partition the training set randomly into 1260 images from the UC Merced Land Use Dataset^41^ and 18,900 images from NWPU-RESISC45^42^. Considering various steganography algorithms and embedding rates, we dedicate 8064 images to each of SUNIWARD^43^ and WOW steganography. By contrast, we allocate 12,096 images to HILL^30^ steganography, as its distortion costs are markedly different from the other two algorithms. On average, each steganography algorithm incorporates five different embedding rates. We augment the data by implementing rotation and transposition transformations. Following the training of SRNet on this dataset, we select cover images guided by the saved pre-trained SRNet models. We favor selecting images that are either more straightforward or more challenging to be evaluated by the pre-trained model as cover images. The entire process is depicted in Fig. 2.

**Figure 3:**
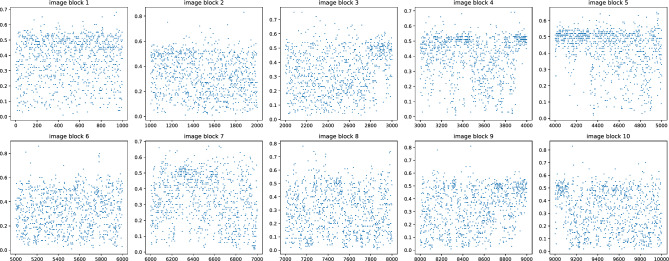
Visualization of the hybrid trained SRNet to judge the stego probability with an embedding rate of 0.3bpp.

As illustrated in Fig. 3, the hybrid pre-trained model SRNet identified the likelihood of stego images. The figure shows the probability of steganography for each image in the dataset, with the UT-GAN algorithm as the steganography method and an embedding rate of 0.3 bpp (bits per pixel). The x-axis represents the image number, while the y-axis represents the probability of steganography. The cover image should be selected from the image corresponding to the figure’s lowest point, as it indicates the most negligible probability of steganography.

### Security-oriented adaptive payload allocation strategy

The conventional approach for payload allocation in multi-image steganography involves evaluating the embedding cost of each pixel in all cover images using a specific stego algorithm. The calculated embedding cost values are sorted and embedded in ascending order to minimize overall embedding distortion. Similarly, secret information is embedded in ascending order to further reduce distortion. However, traditional steganography algorithms have limitations in terms of security in multi-image steganography, while deep learning-based steganography algorithms have improved the security of concealing secret information.

This paper introduces UT-GAN, a pre-trained stego network designed for payload allocation in multi-carrier steganography. UT-GAN is based on the concept of ASDL-GAN^44^ and utilizes a U-Net^45^ generator to convert a cover image into a embedding probability map. The optimal embedding and creation of the final stego image are facilitated by a double-tanh function, which requires no pre-training. To assess the security of UT-GAN, XuNet^46^, an advanced steganalysis network, is employed for high-pass filtering on both cover and stego images. UT-GAN automatically learns the cost of stego distortion by iteratively updating the network parameters, resulting in higher security compared to traditional hand-crafted stego methods. For steganography, UT-GAN utilizes STC^47^, a nearly optimal coding scheme that aims to minimize the embedding distortion cost. The embedding distortion cost measures the extent of change an image undergoes due to steganography, while the embedding modification probability indicates the likelihood of altering a pixel value in the DCT (Discrete Cosine Transform)^48^ domain. The correlation between them is defined as follows:

17 $$\begin{aligned} \rho _i\left( s_i=c_i+1\right) =\rho _i\left( s_i=c_i-1\right) =\ln \left( \frac{1}{\beta _i}-2\right) \end{aligned}$$

As the stego modification probability $\beta _i$ of a pixel increases, the stego distortion cost $\rho _i$ decreases. Consequently, we prioritize pixels with higher modification probabilities for payload allocation, arranging them accordingly.

We propose a security-oriented adaptive payload allocation (SoA-PA) strategy to allocate appropriate payloads on multiple selected cover images. The core concept of this strategy revolves around embedding secret information based on the security level, thereby maximizing the stego security of each image. This approach ensures steganography security at the image level and employs a novel algorithm to optimize the steganographic capacity of each image, thereby enhancing the security of multi-image steganography. To account for the gradual transition from insecure to secure steganography, we introduce a payload tolerance parameter ($\delta$), which determines the maximum payload that can be embedded in an image without compromising security.

**Figure 4:**
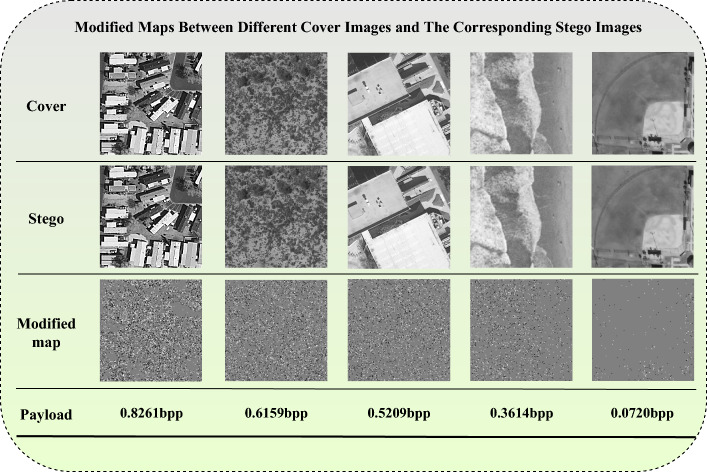
Visualization of our proposed steganography payload allocation results.

Let *N* denote the number of selected images and *M* represent the length of the secret information. Our SoA-PA strategy follows these steps: First, we sort the dataset images in descending order based on their stego security, as measured by the pre-trained SRNet. Second, we initialize the embedding payload of each image to be evenly allocated, ensuring that each image has an embedding payload length of *M*/*N*. This approach guarantees high security even for large payloads. However, it may result in a lower steganographic capacity since the average embedding strategy is more secure than individual image capacity when the average payload exceeds each image’s maximum stego security capacity^49^. Third, starting with the image with the highest security level, we attempt to embed the remaining secret information $R_i$. If the embedding remains undetectable by the pre-trained SRNet, we embed the secret information with a length of $R_i$. Otherwise, we embed half of this amount, $R_i / 2$. The embedding order of all images is determined by the pre-trained UT-GAN model. Fourth, we transform the images into corresponding modification probability maps and embed the modifications in order of probability, from the largest to the smallest^50^. Figure 4 illustrates the modified maps of some cover images^19^.

**Algorithm 1:**
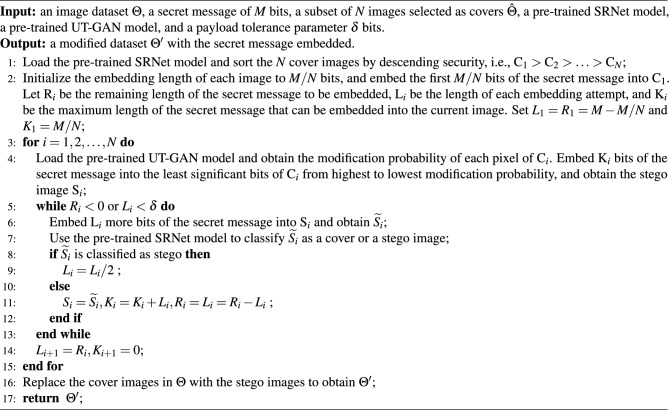
Proposed SoA-PA strategy

The proposed SoA-PA strategy for multiple remote sensing images offers two key advantages. Firstly, it ensures high security while accommodating both small and large payload capacities. This approach maximizes security by embedding secret information within the most robust image, capable of evading SRNet detection. Consequently, it serves as an effective and secure strategy for multi-image steganography payload allocation. When the secret information’s length is less than $\delta$, the embedding process continues by selecting the next image. We strive to embed the entire remaining information length, evaluating the steganalysis model’s detection resistance. This iterative process repeats until all secret information is embedded. Finally, the embedded information replaces the corresponding images from the original dataset. The detailed payload allocation strategy is outlined in Algorithm 1.

**Figure 5:**
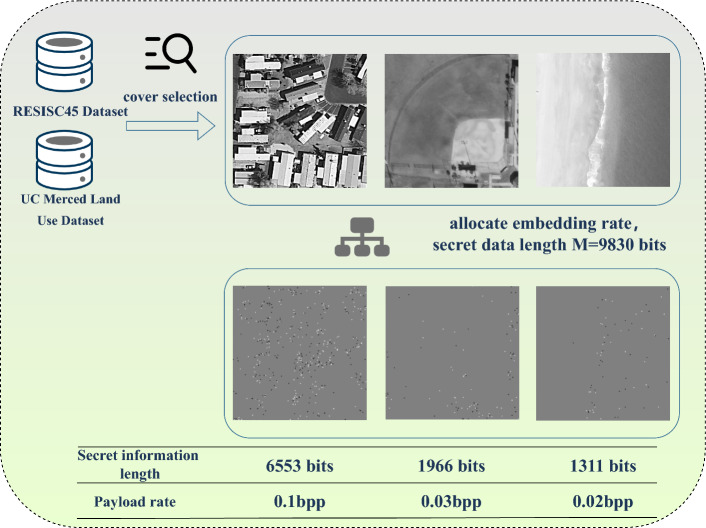
Visualization of payload allocation results.

Figure 5 demonstrates the practical application of SoA-PA strategy combined with cover selection from two databases. We assume that three images are selected, as depicted in the figure, and that the UT-GAN steganography algorithm is used for embedding. We also establish the payload tolerance parameter at $\delta$ = 10 bits and the total embedding length at 9830 bits. Based on SoA-PA strategy, the calculated optimal embedding lengths for the three selected images are 6553 bits, 1966 bits, and 1311 bits, respectively. The corresponding payload rates are 0.1 bpp, 0.03 bpp, and 0.02 bpp.

## Experimental results

### Datasets

We employ two openly accessible datasets containing remote sensing images: NWPU-RESISC45^51^ and UC Merced Land Use Dataset^52^. Table 1 provides an overview of the fundamental characteristics of these datasets. Both datasets encompass natural and human-made scenes, including agricultural, forested, industrial, and residential areas.

**Table 1:** Basic information of the two remote sensing datasets.

Dataset	Classes	Images
NWPU-RESISC45	45	31,500
UC Merced Land Use Dataset	21	2100

We employed stratified random sampling on these datasets to ensure that each class within the sample was proportionally represented. Each dataset was divided into training and testing subsets in an 8:2 ratio, yielding 26,880 training images and 6720 testing images. Data augmentation techniques like flipping and rotation were applied to enhance the diversity and robustness of the training set. To enhance model convergence and diminish the influence of illumination variations, we standardized each image by subtracting the dataset’s mean and dividing it by its standard deviation. Additionally, we converted the images to grayscale to streamline computations and diminish noise. Grayscale images contain a single channel, whereas color images comprise three. To lower computational expenses and guarantee compatibility with the pre-trained model, all images underwent resizing to a resolution of $256 \times 256$ pixels. Using Python, specifically the PIL and OpenCV libraries, we converted the images from TIF and RGB format to PGM format. The PGM format is a standard widely utilized for image processing and analysis.

However, contrary to single-image steganography and analysis, multi-image steganography and analysis security depend on the number of users. Therefore, in this paper, we investigate the effect of the number of users on the security of multi-image steganography by altering the number of users to $n \in \{10,25,50\}$.

### Evaluation metrics

We assess the security of multi-image steganography at both the individual and image levels. At the individual level, we employ the LOF to gauge the level of abnormality for each image within the cover set. At the image level, we utilize the Spatial Rich Model (SRM)^19^ feature set, a widely accepted tool in steganalysis, to quantify the distortions steganography introduces. Alternatively, we can leverage features extracted by SRNet, a deep learning-based steganalysis model, to evaluate image-level security. Given that our proposed adversarial pre-training model is structured around SRNet, we opt for the conventional 34,761-dimensional SRM feature set as the primary criterion for evaluating image-level security.

SRM captures diverse features from image noise residuals, reflecting alterations in local pixel dependencies induced by steganography. SRM employs multiple sub-models to filter the image, acquire distinct residuals, and subsequently compute co-occurrence matrices of these residuals, thereby constructing high-dimensional features. The ensuing formula delineates the feature vector of SRM:

18 $$\begin{aligned} \text{SRM} = \sum _{i=1}^{N} w_i \cdot f_i(x, y) \end{aligned}$$

where $N$ is the number of noise residuals considered. $w_i$ represents the weight of the $i$-th residual. $f_i(x, y)$ is the function that computes the $i$-th residual at pixel location $(x, y)$.

### Safety assessment of cover selection

The use of SRM features to measure the security of various cover selection strategies is highlighted for clarity. The strategies under comparison encompass joint cover selection, minimum distortion selection, and the similarity-based strategy outlined in literature^24^, literature^13^, and literature^14^. The experimental procedure is as follows: We crafted the vector selection in this study through a hybrid training approach involving pre-trained SRNet. Nevertheless, we employed the SRNet explicitly trained for the designated steganography algorithm and embedding rate to ensure equitable comparisons with alternative schemes. Subsequently, we chose 3000 images from the training dataset and employed diverse cover selection strategies for steganography. The steganography algorithms employed consisted of SUNIWARD and HILL, with embedding rates ranging from 0.1 bpp to 0.5 bpp. Optimal simulation embedding was utilized for each scenario. To mitigate the impact of randomness, we conducted ten repetitions of each experiment and computed the average value as the conclusive outcome.

**Table 2:** Average error rate of SRM-based feature detection under various cover selection strategies.

Algorithm	Embedding rate	Randomization strategy	Literature^24^ strategy	Literature^13^ strategy	Literature^14^ strategy	**Ours**
SUNIWARD	0.1	0.4236	0.4388	0.4781	0.4792	**0.4802**
	0.2	0.3481	0.3793	0.4204	0.4277	**0.4319**
	0.3	0.2809	0.3233	0.3836	0.3901	**0.3991**
	0.4	0.2289	0.2756	0.3532	0.3578	**0.3698**
	0.5	0.1917	0.2588	**0.3123**	0.3091	0.3112
HILL	0.1	0.4527	0.4682	0.4851	0.4889	**0.4905**
	0.2	0.3836	0.4382	0.4697	0.4782	**0.4781**
	0.3	0.324	0.3791	0.4257	0.4300	**0.4385**
	0.4	0.2875	0.3278	**0.3968**	0.3955	0.3963
	0.5	0.2402	0.3023	**0.3740**	0.3673	0.3722

Significant values are in bold.

As demonstrated in Table 2, our proposed method consistently attains the lowest average error rate at low embedding rates and maintains competitiveness at higher embedding rates. However, the cover selection approach introduced by Wang et al.^13^ is computationally intensive due to the necessity of calculating and minimizing both steganographic distortion and processing distortion associated with the selected images. Consequently, this renders it unfeasible for multi-image steganography applications. Conversely, acquiring a steganalysis pre-trained model is straightforward and facilitates real-time image analysis. Moreover, Wang et al.^14^ have provided evidence of the impracticality of their technique when handling multi-image stego scenarios with varying embedding rates and steganography algorithms.

Conversely, our approach can manage such scenarios by fusing embedding with a pre-trained dataset, which can be expanded to enhance cover selection accuracy. In summary, we introduce a more efficient and viable strategy for cover selection in the context of multi-image steganography. Our approach surpasses others in terms of performance and practical utility.

### Security assessment of stego-individuals

This paper introduces a novel steganographic strategy for application in multiple remote sensing images. It assesses the security of this strategy utilizing the LOF. The primary objective of this strategy is to reduce both the quantity and distortion of stego images while maximizing their capacity and security. The paper conducts a comparative analysis of the newly proposed strategy against three existing approaches: ES-UPD, ES-ITC, and ES-DD^11^.

ES-UPD evenly allocates the payload across all images without considering their characteristics. In contrast, ES-ITC evaluates the capacity of each image using entropy and chooses the most suitable candidates for steganographic purposes. On the other hand, ES-DD allocates the payload among all images based on their distortion cost, which is calculated using a pre-trained SRNet. The newly proposed strategy amalgamates and amplifies the advantages of ES-ITC and ES-DD. Specifically, it employs SRNet for estimating the capacity of individual images, a more dependable approach than entropy-based estimation. Additionally, it adopts a sequential approach to embed the payload into images, prioritizing their security and modification probability. This results in a reduced number of stego images in comparison to ES-DD. The study utilizes the UT-GAN algorithm for steganography, employing five different embedding rates ranging from 0.1 to 0.5 bpp. Subsequently, after embedding the payload within a set of images, the paper proceeds to extract SRM features from all images and compute the MMD distance for each pair of images. The subsequent step involves the computation of LOF values for each image, derived from their MMD distances about others. The image exhibiting the highest LOF value is identified as the steganographer. Each configuration is iterated ten times to ensure experimental validity, and the mean outcome is subsequently reported.

**Table 3:** UT-GAN steganography combined with LOF values and rankings under the SoA-PA strategy in this paper.

LOF ranking	0.1 bpp	0.2 bpp	0.3 bpp	0.4 bpp	0.5 bpp
1	1.2392	1.2426	1.2449	1.2431	1.2193
2	1.1216	1.1319	1.1337	1.1391	1.2004
3	1.1171	1.1203	1.1226	1.124	1.1932
4	1.1169	1.1203	1.1226	1.1229	1.1137
5	1.1044	1.1052	1.1093	1.1229	1.1137
6	1.0873	1.0942	1.1012	1.1092	1.0979
7	1.0312	1.0327	1.0361	1.038	**1.0083**
8	**1.0041**	**1.0005**	**0.9989**	**0.9977**	0.9691
9	0.9662	0.9677	0.9701	0.9716	0.9552
10	0.8911	0.893	0.8955	0.8972	0.8817

Significant values are in bold.

**Figure 6:**
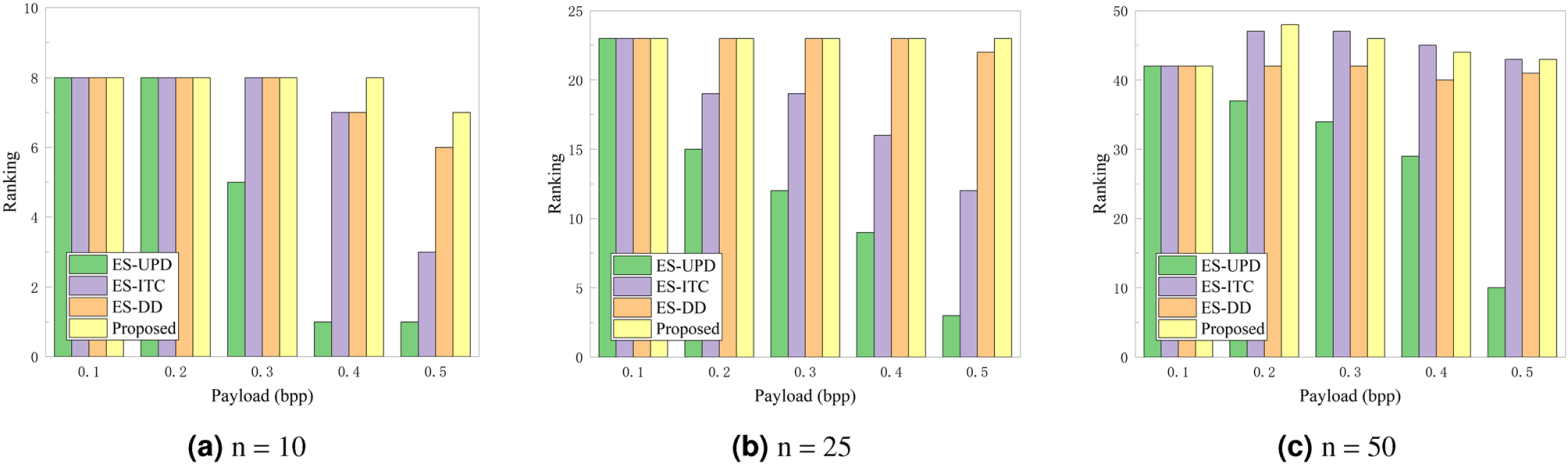
Impact of different number of individuals on LOF rankings.

Table 3 presents the LOF values and rankings for each individual, as determined by our proposed SoA-PA strategy, within the context of the UT-GAN steganography algorithm. This analysis is conducted with a cohort of $n=10$ individuals. The LOF values corresponding to the steganographers are highlighted in bold, with higher rankings indicating a greater likelihood of detection. Notably, our method yielded substantial LOF rankings, specifically 8, 8, 8, 8, and 7, across the five different payloads, underscoring a commendable level of security. Nevertheless, it is essential to note that the LOF rankings exhibited variability when we modified the payload allocation strategy while maintaining all other conditions constant, as demonstrated in Fig. 6a. To further explore the impact of the number of individuals on the LOF rankings, we conducted additional experiments with $n=25$ and $n=50$, with the outcomes depicted in Fig. 6b,c, respectively.

An appropriate allocation strategy can enhance security at the individual level within multi-image steganography. Nonetheless, the uniform allocation strategy denoted as ES-UPD experiences a decline in security as the payload escalates. This strategy uniformly incorporates information across all images without regard for individual distinctions. Consequently, this approach necessitates minimal payloads to sustain a stable level of security. Beyond this threshold, security experiences a substantial decline as the payload surpasses the secure steganographic capacity of numerous images. Otherwise, security deteriorates significantly as the payload exceeds the secure steganographic capacity of many images.

In contrast, our proposed method consistently attains elevated LOF rankings and maintains strong security across various population sizes. Our approach amalgamates the merits of ES-ITC and ESDD strategies to enhance security at the individual level in multi-image steganography. This is achieved by embedding a minimal number of secure cover images in accordance with the stego modification probability. Consequently, our method effectively safeguards security at the individual level in the context of multi-image steganography.

### Security assessment of stego images

To ascertain the identity of the steganographer precisely, the researchers introduced a novel methodology. They advocated that to augment the security of multi-image steganography; it is imperative to focus on security considerations at both the individual and image levels. This entails that after applying steganography, the steganographer should engage in a stego analysis of the image, aiming to bolster its resilience against steganalysis. The higher the resistance, the more secure the image is.

Our study utilized a training dataset comprising 26,880 images from the NWPU-RESISC45 and UC Merced Land Use Dataset. The deep steganalysis network could apply rotations and transpositions to the dataset, enhancing its detection capabilities. Significantly, these operations did not alter the symmetry of SRM features. Three steganography algorithms, namely SUNIWARD, HILL, and UT-GAN, were employed, each at five distinct embedding rates spanning from 0.1bpp to 0.5bpp. Furthermore, a comparative analysis was conducted on three payload allocation strategies: ES-UPD, ES-ITC, and ES-DD^11^.

The efficacy of these diverse strategies was evaluated by quantifying the error rates in steganalysis across various groups in comparative experiments. The results are presented in Table 4. Subsequently, the ensuing section outlines experiments designed to assess the security of multi-image steganography at the image level, utilizing two prevalent steganalysis techniques.

**Table 4:** Steganography detection error rate combining four payload allocation strategies with three steganography algorithms^11^.

Algorithms	Embedding rate (bpp)	SRM				SRNet			
		ES-UPD	ES-ITC	ES-DD	**Ours**	ES-UPD	ES-ITC	ES-DD	**Ours**
SUNIWARD	0.1	0.4226	**0.4481**	0.4464	0.4477	0.3094	0.3126	0.3292	**0.3327**
	0.2	0.3418	0.3942	0.4027	**0.414**	0.2113	0.2207	0.2537	**0.2611**
	0.3	0.2769	0.3499	0.3765	**0.3851**	0.1469	0.1592	0.2015	**0.2203**
	0.4	0.2281	0.2911	0.2713	**0.3119**	0.1059	0.1086	0.1662	**0.1829**
	0.5	0.1864	0.2617	0.2035	**0.2683**	0.0683	0.07	0.1093	**0.1326**
HILL	0.1	0.4562	**0.4852**	0.4771	0.4803	0.3155	0.3204	0.3417	**0.3492**
	0.2	0.3834	0.4434	0.4526	**0.4655**	0.2349	0.2426	0.3011	**0.315**
	0.3	0.3212	0.3925	0.4213	**0.4394**	0.1826	0.1885	0.2796	**0.2837**
	0.4	0.2811	0.3705	0.3752	**0.3881**	0.1405	0.1451	0.2324	**0.2455**
	0.5	0.2375	0.3391	0.3407	**0.349**	0.1147	0.1165	0.199	**0.2076**
UT-GAN	0.1	0.4414	**0.4696**	0.4602	0.4637	0.3572	0.3647	0.3972	**0.4188**
	0.2	0.3825	0.4104	0.4321	**0.4413**	0.3027	0.3155	0.3531	**0.3605**
	0.3	0.336	0.3902	0.4116	**0.4292**	0.2664	0.271	0.3375	**0.3514**
	0.4	0.2951	0.3211	0.3289	**0.3427**	0.2219	0.2253	0.2676	**0.2792**
	0.5	0.2417	0.3046	0.3118	**0.3125**	0.188	0.1889	0.2349	**0.2443**

Significant values are in bold.

We adapted the initial payload allocation strategy using SUNIWARD or HILL steganography within the experimental framework outlined in this manuscript. Rather than embedding modification probabilities derived from UT-GAN in descending order of probability, we opted to embed the respective stego algorithms following descending order of embedding cost, as explicitly delineated in Algorithm 1. Our algorithm exhibited superior performance to ES-ITC regarding steganography detection error rate across all configurations, except for the setting at 0.1bpp when utilizing SRM features. Furthermore, our strategy demonstrated the highest level of image-level security when employing SRNet for steganalysis.

The degree of enhancement achieved by ES-ITC was considerably less pronounced when compared to SRM detection and ESDD. Nonetheless, our strategy continued to exhibit a noteworthy enhancement effect. This can be attributed to the fact that SRNet can capture more intricate steganalysis features and is less influenced by the image’s content, in contrast to traditional SRM features. Specifically, under the HILL steganography algorithm, and at an embedding rate of 0.3bpp, our payload allocation strategy resulted in a 10.5% increase in the steganography detection error rate compared to the average allocation. This noteworthy enhancement in the security level of multiple stego images underscores the exceptional performance of our strategy.

In summary, our SoA-PA strategy effectively enhances the security of multi-image steganography at the image level.

### Assessment of remote sensing images

Remote sensing images exhibit attributes such as high resolution, dynamic range, spectral richness, and significant spatial and temporal resolution. These characteristics suggest that they possess the potential to provide superior information capacity and heightened security in practical applications when compared to general images. Consequently, we posit the hypothesis that remote sensing images represent a more favorable choice as steganographic cover. In order to substantiate our hypothesis, we conducted a series of experiments to evaluate the security and steganographic capacity of remote sensing image steganography.

#### Security Assessment of Remote Sensing Images

We utilized two datasets, namely BOSSBase^53^ and NWPU-RESISC45, for conducting the security assessment of remote sensing images. BOSSBase is a well-known dataset in the field of image steganography, consisting of 10,000 grayscale images in a $512 \times 512$ PGM format. The BOSSBase images were resized to dimensions of $256 \times 256$, followed by a random selection of 1000 images from each dataset. Subsequently, an SRNet was trained on a randomly selected subset of images from both datasets, using the same methodology as previously described. The test images were sorted according to their steganographic capacity, followed by the application of the UT-GAN, SUNIWARD, and HILL algorithms for multi-image steganography to both categories of images. These algorithms were utilized with embedding rates ranging from 0.1bpp to 0.5bpp based on our proposed strategy. The detection of stego images was conducted using both SRM and SRNet, and the resulting error rates were meticulously documented.

**Table 5:** Steganography detection error rate on NWPU-RESISC45 and BOSSBase with three steganography algorithms.

Datasets	Embedding rate (bpp)	SRM			SRNet		
		SUNIWARD	HILL	UT-GAN	SUNIWARD	HILL	UT-GAN
NWPU-RESISC45	0.1	0.4400	0.4588	**0.4768**	0.3240	0.3468	**0.4359**
	0.2	0.3946	0.4379	**0.4599**	0.2739	0.2977	**0.3810**
	0.3	0.3611	0.3793	**0.4076**	0.2567	0.2643	**0.3495**
	0.4	0.2910	0.3544	**0.3631**	0.1907	0.2370	**0.2861**
	0.5	0.2667	0.3084	**0.3357**	0.1466	0.2054	**0.2466**
BOSSBase	0.1	0.3601	0.3803	**0.3971**	0.2186	0.2367	**0.2864**
	0.2	0.3209	0.3468	**0.3598**	0.1804	0.2033	**0.2504**
	0.3	0.2855	0.3026	**0.3344**	0.1465	0.1804	**0.2306**
	0.4	0.2321	0.2677	**0.2737**	0.1137	0.1476	**0.1824**
	0.5	0.2168	0.2130	**0.2384**	0.0947	0.1243	**0.1587**

Significant values are in bold.

The data presented in Table 5 illustrates that the detection error rates for general images exceed those of remote sensing images. This discrepancy suggests that remote sensing images exhibit higher steganographic security. This phenomenon can be attributed to the intricate texture features inherent to remote sensing images, which render the detection of anomalies by steganalysis more challenging.

#### Capacity assessment of remote sensing images

We implemented our proposed steganographic payload allocation strategy to substantiate the assertion that remote sensing images possess superior steganographic capacity compared to general images. Images sourced from BOSSBase and NWPU-RESISC45 were meticulously arranged based on their steganographic security ranking, a metric derived from the hybrid pre-trained SRNet. Subsequently, a fixed-length binary secret message consisting of 1,000,000 bits was systematically embedded into the cover images, utilizing a sequential approach. Three distinct steganographic algorithms, namely SUNIWARD, HILL, and UT-GAN, were employed for this purpose. The maximum steganographic capacity was allocated to each cover image, and the embedding process ceased either when all the available images were utilized or when the entire message had been embedded. This experimentation was repeated ten times, and data regarding the average and coefficient of variation in the number of cover images required for both categories of images were meticulously documented. Table 6 provides an overview of the results.

**Table 6:** Quantities of cover images required to embed a secret message of the same length according to the three steganographic algorithms of the SoA-PA strategy proposed in this paper.

Datasets	SUNIWARD		HILL		UT-GAN	
	Mean	CV	Mean	CV	Mean	CV
NWPU-RESISC45	36.7	0.09	34.0	0.12	31.5	0.07
BOSSBase	54.2	0.13	52.7	0.17	48.5	0.10

The empirical evidence, as presented in Table 5, supports the assertion that remote sensing images can embed more secret messages while utilizing fewer cover images than general images. This observation underscores that remote sensing images possess an elevated steganographic capacity and exhibit greater efficiency in steganographic operations. This phenomenon can be attributed to their heightened resolution, expanded dynamic range, and increased grey level, which collectively provides an augmented capacity for information and enhanced noise tolerance. Furthermore, it is confirmed that remote sensing images serve as superior steganographic covers due to their intricate textures and information-rich nature, making detecting embedding changes considerably more challenging.

### Capacity assessment of payload allocation strategies

This subsection evaluates the performance of the SoA-PA strategy by comparison with three existing strategies: ES-UPD, ES-ITC, and ES-DD. These payload allocation strategies integrate three steganography algorithms: S-UNIWARD, HILL, and UT-GAN. Utilizing the hybrid-trained SRNet, cover images in the NWPU-RESISC45 dataset are ranked by security level, with information embedded from the most to the least secure images. A random 1,000,000 bits of information are generated and embedded into the cover images. The number of images required to embed the entire set of information at different embedding levels is recorded, and the experiment is repeated 10 times. The coefficient of variation for the 10 data sets is calculated, with results presented in Table 7.

**Table 7:** Quantities of cover images required to embed a secret message of the same length according to the three steganographic algorithms of ES-UPD, ES-ITC, ES-DD and our SoA-PA strategy.

Algorithms	Embedding	ES-UPD		ES-ITC		ES-DD		**Ours**	
	rate (bpp)	Mean	CV	Mean	CV	Mean	CV	Mean	CV
SUNIWARD	0.1	156.7	0.11	156.6	0.09	155.9	0.07	**152.9**	0.03
	0.2	81.2	0.12	80.5	0.08	80.6	0.08	**76.5**	0.04
	0.3	54.5	0.09	53.6	0.10	53.7	0.08	**51.1**	0.04
	0.4	43.6	0.11	42.8	0.07	43.0	0.09	**38.4**	0.05
	0.5	35.9	0.12	35.1	0.08	34.9	0.07	**30.8**	0.04
HILL	0.1	155.4	0.10	155.6	0.09	155.2	0.08	**152.9**	0.02
	0.2	79.9	0.08	79.4	0.07	79.7	0.10	**76.6**	0.04
	0.3	54.1	0.08	53.2	0.06	53.5	0.12	**51.0**	0.03
	0.4	42.4	0.07	42.1	0.08	42.2	0.09	**38.3**	0.05
	0.5	35.3	0.14	34.6	0.10	34.7	0.12	**30.9**	0.04
UT-GAN	0.1	154.6	0.13	155.0	0.08	154.8	0.12	**152.8**	0.03
	0.2	80.4	0.07	79.7	0.07	79.6	0.10	**76.4**	0.02
	0.3	53.8	0.11	53.1	0.07	53.2	0.13	**51.1**	0.04
	0.4	42.1	0.10	41.6	0.08	41.8	0.11	**38.5**	0.03
	0.5	34.8	0.12	34.5	0.11	34.4	0.11	**30.9**	0.05

Significant values are in bold.

Experimental findings indicate that the SoA-PA strategy implements a highly flexible steganography embedding approach. By consistently aiming to approximate the specified embedding rate, it ensures embedding efficiency even as the embedding rate level increases. Concurrently, experimental findings reveal that the SoA-PA strategy accomplishes steganography objectives using fewer cover images, concurrently safeguarding the security of steganography images. Moreover, the utilization of fewer stego images enhances steganography security, further affirming the reliability of the proposed method.

### Computational complexity analysis

This section analyzes the computational complexity of the payload allocation strategy. The SoA-PA strategy employs a pre-trained UT-GAN, an unsupervised texture transformation model based on generative adversarial networks. During the inference process, UT-GAN encodes and decodes each pixel block, in contrast to S-UNIWARD, HILL, and WOW, which are spatial steganography methods. Calculating the cost function for each pixel renders these methods particularly time-consuming, especially for high-resolution images. Conversely, UT-GAN circumvents the need for cost function calculation by utilizing the modification probability to guide the embedding process, thereby enhancing efficiency. The computational complexity of the SoA-PA strategy can be estimated as follows:

19 $$\begin{aligned} O(N \times (K_{\max } + L_{\max } \times \log L_{\max } + U)) \end{aligned}$$

where *N* denotes the number of selected cover images, $K_{\max }$ indicates the maximum payload size for each image, $L_{\max }$ denotes the maximum number of trials for each embedding, and *U* signifies the sum of the complexities of the SRNet and the UT-GAN. This complexity represents an upper bound, as the actual payload size can be smaller than $K_{\max }$, and the number of trials is reduced by half until reaching the distortion threshold $\delta$ or a successful embedding is achieved.

To quantify these results, experiments were conducted using 1,000,000 bits of information as the message to be embedded. The time required to embed this message was measured for the proposed SoA-PA strategy and three existing strategies (ES-UPD, ES-ITC, and ES-DD). The embedding rate for the ES-UPD, ES-ITC, and ES-DD strategies was set at 0.5 bpp to minimize the number of cover images required, thus diminishing potential time errors associated with reading the images, despite the minimal read time involved. To ensure a fair and reliable comparison, the same order of cover images was used for each embedding, selected by the hybrid-trained SRNet according to the steganography security ranking. The experiment was repeated ten times, and both the mean and the coefficient of variation of the time required were recorded. Table 8 presents the time (in seconds) required by the four strategies to embed the same message length using the four steganographic algorithms.

**Table 8:** Running time combining four payload allocation strategies with four steganography algorithms.

Strategies	SUNIWARD		HILL		WOW		UT-GAN	
	Mean	CV	Mean	CV	Mean	CV	Mean	CV
ES-UPD	4.92s	0.14	2.65s	0.11	2.89s	0.15	7.01s	0.13
ES-ITC	15.02s	0.12	8.69s	0.09	9.64s	0.08	19.62s	0.12
ES-DD	32.77s	0.13	20.56s	0.10	21.48s	0.13	47.56s	0.11
**Ours**	13.79s	0.14	7.47s	0.11	8.93s	0.12	18.23s	0.10

The results demonstrate that the SoA-PA strategy surpasses the other strategies in terms of speed. The computational complexity of this strategy is significantly influenced by the processing speeds of SRNet and UT-GAN, both of which are augmented by the CUDA acceleration module. Unlike the uniform embedding strategy of ES-UPD, our strategy opts for a trade-off between speed and enhanced steganographic security, making it more suitable for real-world applications.

## Conclusion

This paper proposes a novel and practical security-oriented steganographic payload allocation method for multi-remote sensing images. To tackle the issues of cover selection and payload allocation in the context of multi-image steganography, our method creates a direct link between an image and its steganographic security through a steganalysis pre-training model, and a steganography pre-training model is utilized to determine the order of embedding. Furthermore, a payload adaptive allocation scheme is designed to make each image embed secret information as close as possible to the length of the maximum steganographic capacity under the premise of security. By comparing with the existing cover selection strategies and payload allocation methods, the experimental results show that the proposed method multi-image steganography method is generally superior to the state-of-the-art methods and does not require extensive computation, making it well suited for real-world applications.

## Data Availability

The data used in this study are from two publicly available remote sensing image datasets: the UC Merced Land Use Dataset, NWPU-RESISC45 Dataset, and the BOSSBase Dataset. UC Merced Land Use Dataset can be downloaded from this website http://weegee.vision.ucmerced.edu/datasets/landuse.html. NWPU-RESISC45 Dataset is a publicly available benchmark for remote sensing image scene classification. The dataset can be downloaded from OneDrive https://1drv.ms/u/s!AmgKYzARBl5ca3HNaHIlzp_IXjs after filling out a data request form. BOSSBase Dataset can be downloaded from the website http://dde.binghamton.edu/download/ImageDB/BOSSbase_1.01.zip. The code used for data processing and analysis is available from the corresponding author upon request.
